# Impact of Fungi Co-occurrence on Mycotoxin Contamination in Maize During the Growing Season

**DOI:** 10.3389/fmicb.2019.01265

**Published:** 2019-06-06

**Authors:** Paola Giorni, Terenzio Bertuzzi, Paola Battilani

**Affiliations:** ^1^Department of Sustainable Crop Production (Di.Pro.Ve.S), Università Cattolica del Sacro Cuore, Piacenza, Italy; ^2^Department of Animal Science, Food and Nutrition (DIANA), Università Cattolica del Sacro Cuore, Piacenza, Italy

**Keywords:** maize, *Fusarium verticillioides*, *Fusarium graminearum*, *Aspergillus flavus*, aflatoxin, fumonisin, deoxynivalenol

## Abstract

Maize is a possible host of many fungi, some of them able to produce different mycotoxins. Few studies exist on co-occurring fungi and resulting multi-mycotoxin contamination in field; for this reason, in field trials were conducted in two consecutive years to verify fungal incidence and mycotoxin production in the case of the co-occurrence of the three main mycotoxigenic fungi of maize in Italy: *Aspergillus flavus, Fusarium verticillioides*, and *Fusarium graminearum* able to produce, respectively, aflatoxin B1 (AFB1), fumonisins (FBs), and deoxynivalenol (DON). Artificial inoculation was done after silk emergence of maize and samples were collected with a 2 week schedule up to harvest time (four samplings). Fungal interaction resulted as playing a role for both fungal incidence and mycotoxins production, as did weather conditions too. Main interactions were noted between *A. flavus* and *F. verticillioides*, and between *F. verticillioides* and *F. graminearum*. In particular, as a result of fungal co-occurrence, AFB1 resulted stimulated by *F. graminearum* presence while no effects were noted in FBs and DON in case of *F. verticillioides*–*F. graminearum* co-occurrence. Interestingly, the co-presence of *A. flavus* significantly reduced both FB and DON production.

## Introduction

Maize is an important world-wide crop prone to different fungal colonization both in field and during storage. In particular, maize can be a good substrate for some of the best known mycotoxigenic fungi such as *Aspergillus flavus, Fusarium verticillioides*, and *Fusarium graminearum* able to produce, respectively, some of the most hazardous substances for humans and animals: aflatoxins (AFs), fumonisins (FBs), and deoxynivalenol (DON). The environment during in field cultivation or in post-harvest determine the conditions in which fungal species are more likely to develop (Queiroz et al., 2012). However, many fungal species can co-occur and impact each other on growth and mycotoxin production. A complex mixture of fungal metabolites may then contaminate maize (Grenier and Oswald, 2011). Literature report several examples of microbe interactions underlining how fungal metabolites may act as protection against other microbes or contribute for an environmental niche more suitable for their development (Venkatesh and Keller, 2019). In particular, some studies seem to give to mycotoxin an important and direct role in microbial competition for space and nutrients acting as antibacteria (Spraker et al., 2018) while others suggest they can increase pathogenicity of fungi on the host (Jung et al., 2018; López-Díaz et al., 2018). Interestingly, a role of mycotoxins in host defense responses modulation, acting as signaling molecules, is also suggested (Khan and Doohan, 2009; Diamond et al., 2013; Mousa et al., 2015).

Few studies exist on fungal co-occurrence in maize during the growing season and they are limited to the definition of natural occurrence of different mycotoxins in maize without considering fungal presence and/or interactions under in field conditions (Kovalsky et al., 2016; Culig et al., 2017; Oliveira et al., 2017).

Environmental factors, in particular temperature and water activity (a_w_), play a fundamental role in determining fungal species prevalence influencing both their growth and mycotoxins production (Magan and Medina, 2016). Commonly, optimal environmental conditions for fungal growth are different from those considered optimal for mycotoxins production and this is peculiar for each fungal species. *A. flavus* shows optimal growth between 30–35°C (Abdel-Hadi et al., 2012) while *F. verticillioides* and *F. graminearum* grow better, respectively, between 25–30°C (Medina et al., 2013) and 15–25°C (Hope et al., 2005), all with optimal a_w_ = 0.99–0.98. Regarding mycotoxin production, optimal temperature and a_w_ changes: in particular, AFs are produced between 25–35°C in the a_w_ range 0.99–0.95 (Abdel-Hadi et al., 2012), FBs are produced between 20–25°C and 0.98 a_w_ (Medina et al., 2013) while DON is produced between 15–25°C and 0.99–0.97 a_w_ (Hope et al., 2005). All this information comes from *in vitro* studies.

Therefore this study was managed in field with the aim of: (i) monitor the behavior of artificially inoculated *A. flavus, F. verticillioides*, and *F. graminearum* in maize in Northern Italy during the growing season and (ii) describe the impact of their co-occurrence on mycotoxin contamination in maize grain.

## Materials and Methods

In a commercial maize crop (Food and Agriculture Organization-FAO class 700 Hybrid) a field trial was organized to study the dynamic of *A. flavus, F. verticillioides*, and *F. graminearum* after ear artificial inoculation/co-inoculation. It was managed in two consecutive years (2016 and 2017) in Piacenza, northern Italy.

### Inoculum Preparation

One strain of *A. flavus* [ITEM (Istituto Tossine e Micotossine) 8069], one strain of *F. verticillioides* (ITEM 10027), and 1 strain of *F. graminearum* [MPVP (Micoteca Patologia Vegetale Piacenza) 309], able to produce, respectively, AFs, (B_1_ and B_2_) fumonisins (B_1_, B_2_, and B_3_) and DON and stored in the official fungal collection of the Institute of Sciences of Food Production of the National Research Council (ISPA-CNR) in Bari and/or in the fungal collection of the Department of Sustainable Crop Production (Di.Pro.Ve.S.) of the Università Cattolica del Sacro Cuore in Piacenza, were used for inoculum preparation.

The strains were singularly inoculated on Petri dishes (Ø 9 cm) with Potato Dextrose Agar (PDA, Biolife, Milano, Italy) and incubated at 25°C for 7 days (12 h light/12 h dark photoperiod). At the end of incubation, the dishes were washed with 10 mL of sterile distilled water. The obtained suspension of each fungus was adjusted to a concentration of 10^5^ spores/mL using a Burker chamber for spore count.

### Inoculation of Maize Ears

A total of seven treatments were considered in field: (1) *A. flavus* alone, (2) *F. verticillioides* alone, (3) *F. graminearum* alone, (4) *A. flavus* + *F. verticillioides*, (5) *A. flavus* + *F. graminearum*, (6) *F. verticillioides* + *F. graminearum*, and (7) *A. flavus* + *F. verticillioides* + *F. graminearum*. For maize inoculation single fungus suspensions, prepared as previously described, were used. Twenty maize ears were inoculated for each treatment after silk emergence (25th July in 2016 and 21st July in 2017). Pin bar inoculation, previously suggested as the most appropriate method (Giorni et al., 2016), was applied deepening a three-needle fork into a single fungus inoculum suspension and puncturing the ear in the central part. In case of inoculation with two or three fungal strains, the fork was disinfected with absolute ethanol, rinsed with sterilized distilled water and deepened in the second or third single fungus suspension and then applied on the same wounds formed on the ear by the previous inoculation. Inoculated plants of each thesis were separated by two rows of untreated plants from those of other treatments. Control maize plants, not inoculated, were also included in the study.

Five maize ears for each treatments were collected from early dough to maize harvest (4 sampling times; 10–15 day schedule from artificial inoculation – DAI).

### Treatment of Samples

After husk elimination, maize ears ideally shared in three parts, upper, central (where pin bar inoculation occurred) and lower. Ears were hand shelled keeping only kernels from the central part; 50 kernels were randomly chosen, surface disinfected and transferred on Petri dishes containing PDA. After incubation at 25°C for 5–7 days (12 h light photoperiod), kernels infected by fungi, intended as kernels showing a growing fungal colony after incubation (total fungi) were counted in all the replicated thesis; the incidence of total fungi was calculated rating the counted colonies on 50, the number of plated kernels. The incidence of previously mentioned fungal sections was also calculated following the same approach.

All kernels coming from the central area of each ear and not plated for fungi isolation were used for mycotoxin analysis.

Fungi were identified at section level as *Aspergillus* section *Flavi* (*AsF*) according to Raper and Fennell (1965), *Gibberella fujikuroi* species complex (*Gfsc*) and *F. graminearum* species complex (*Fg*sc) according to Summerell et al. (2003).

Water activity of maize ears was measured in both year at each sampling time using AquaLab Pre (Meter Food, Pullman, WA, United States).

### Mycotoxins Analysis

The kernels were dried at 65°C for 2 days, milled using a cyclone hammer mill (1 mm sieve) (Pulverisette, Fritsch GmbH, Idar-Oberstein, Germany) and homogenized. Three out of five replicates for each thesis were considered for mycotoxin determination.

Analyses and standard preparations were performed according to the methods reported by Bertuzzi et al. (2012) for AFs, by Pietri and Bertuzzi (2012) for FBs and Bertuzzi et al. (2014) for DON. Briefly, AFB1 was extracted using acetone:water 7+3 v/v and purified trough immuno-affinity column (R-Biopharm Rhône LTD, Glasgow, Scotland, United Kingdom); then, the mycotoxin was determined by a HPLC (High Performance Liquid Chromatography) instrument with fluorescence detector. Chromatographic separation was carried out on a Superspher RP-18 column (4 μm particle size, 125 × 4 mm i.d., Merck) at ambient temperature with a mobile phase water-methanol-acetonitrile (64+23+13, v/v/v). AFB_1_ were detected after post-column photochemical derivatization (UVE, LCTech GmbH, Dorfen, Germany); the fluorimeter was set at 365 nm excitation and 440 nm emission wavelengths. The limit of detection (LOD) and the limit of quantification (LOQ) were 0.05 and 0.15 μg/kg, respectively. After extraction with phosphate buffer and purification through immuno-affinity column (R-Biopharm Rhône LTD), FBs were quantified by a HPLC-MS/MS (High Performance Liquid Chromatography coupled with mass spectrometer) system. FBs were separated on a Betasil RP-18 column (5 μm particle size, 150 × 2.1 mm, Thermo Fisher Scientific) with a mobile-phase gradient acetonitrile-water (both acidified with 0.2% formic acid) from 25:75 to 55:45 in 9 min, then isocratic for 3 min; gradient to 75:25 in 1 min and isocratic for 3 min (wash-step). The ionization was carried out with an ESI interface (Thermo Fisher Scientific) in positive mode as follows: spray capillary voltage 4.5 kV, sheath and auxiliary gas 35 and 14 psi, respectively, temperature of the heated capillary 270°C. For fragmentation of [M+H]^+^ ions (722 m/z for FB_1_ and 706 m/z for FB_2_), the argon collision pressure was set to 1.5 m Torr and the collision energy to 36 V. The selected fragment ions were: 704, 352, and 334 m/z for FB_1_, 688, 336, and 318 m/z for FB_2_. The LOD and the LOQ were 10 and 30 μg/kg, respectively. Finally, DON and NIV were extracted with acetonitrile:water 86:14 v/v, purified through a Trilogy-Puritox Trichothecenes column (R-Biopharm Rhône LTD) and quantified by GC-MS. Diacetoxyscirpenol (DAS) was used as internal standard. The PTV temperature was raised from 70°C (held 0.2 min) to 260°C (held for 2 min) at 10°C⋅s^-1^. The oven temperature programming was from 125°C (held for 1 min) to 245°C at 10°C⋅min^-1^ and then to 300°C (held for 1 min) at 30°C⋅min^-1^. MS transfer-line and ion source temperature were at 230°C and 250°C, respectively. Electron ionization at 70 eV and selected ion monitoring (SIM) were used for detection. Fragment ion peaks monitored were 393, 407, 422, and 512 for DON, 377, 392, 407, 467, 510, and 585 for NIV, 350, 377, and 392 for DAS. The LOD and LOQ were 10 and 30 μg/kg, respectively. All the recoveries were higher than 90%.

### Meteorological Data

A meteorological station was chosen close to the maize field and data on temperature (°C) and rain (mm) were collected hourly during the period 1st April–30th September for both the years considered in the study.

### Data Analysis

Data on fungal incidence were arcsine transformed, while mycotoxin content in flour was ln transformed before statistical analysis (Fowler and Cohen, 1990; Clewer and Scarisbrick, 2001).

All data obtained were subjected to univariate analysis of variance (ANOVA) using the generalized linear model (GLM) procedure and significant differences between means were confirmed using Tukey test.

The statistical package IBM SPSS statistics 25 (IBM Corp., Armonk, NY, United States) was used for data analysis.

## Results

### Meteorological Data

**FIGURE 1:**
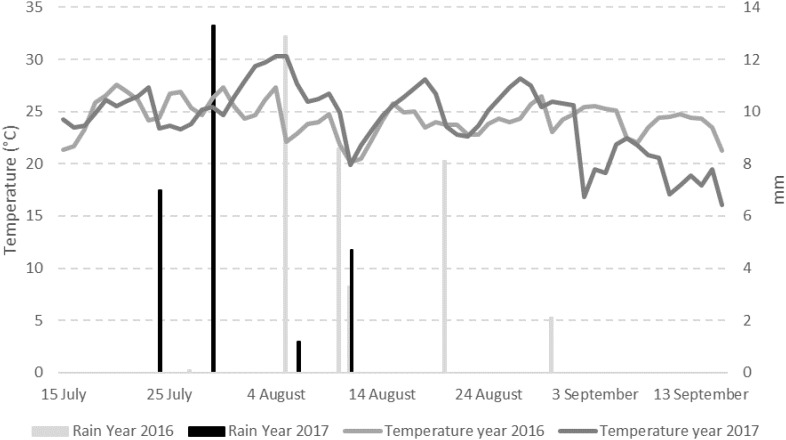
Mean daily data of temperature (°C) and rain (mm) registered in the field used for the trials in the period 1st April–30th September in years 2016 and 2017.

The weather conditions recorded during the 2 years considered in the study resulted different with year 2017 slightly cooler but drier than 2016 (Figure 1). The total sum of temperatures was 1595°C in year 2016 and 1443°C in year 2017 while the total sum of rain resulted to be 36.9 mm in the first year and 27.9 mm in the second year, with 7 and 4 rainy days respectively (Table 1). Consequently, mean relative humidity (RH) was lower in year 2017 than in year 2016 (54.3 vs. 59.3%) (Table 1).

**Table 1:** Temperatures and rainfall occurring in years 2016 and 2017 considering mean daily temperature (°C), sum of temperatures (Σ T), progressive sum of temperature, mean relative humidity (%), total rainfall (Σ Rain – mm), and days w-sith rain in different 2-week periods starting 14 days before artificial inoculation (DAI).

	Year	Mean T (°C)	Σ T	Progressive Σ T	Mean RH (%)	Σ Rain (mm)	Days with rain
14 days pre-inoculation	2016	25.2	378.4	378.4	55.5	1.8	1
	2017	25.7	386.1	386.1	54.1	8.7	1
14 DAI	2016	25.3	354.1	732.5	60.3	13.0	2
	2017	24.9	249.4	635.5	57.0	13.3	1
28 DAI	2016	23.3	233.5	966.0	58.0	11.9	2
	2017	28.2	253.8	889.3	56.7	1.2	1
42 DAI	2016	24.0	312.4	1278.4	62.3	10.2	2
	2017	24.1	193.1	1082.4	53.0	4.7	1
56 DAI	2016	24.4	316.6	1595.0	57.6	0	0
	2017	25.7	360.4	1442.8	50.7	0	0


Regardin a_w_ of maize ears, difference between control and treated plants was not relevant (±0.002); therefore, only a_w_ of non-inoculated ears was considered. Different meteorological conditions caused differences in *aw* level registered in maize kernels during the growing season (Table 2). In particular, in 2016 maize kernels resulted wetter at harvest than in 2017 (0.914 vs. 0.884).

**Table 2:** Mean *aw* level registered in maize kernels of untreated ears collected at different sampling times during the maize growing season in the 2 years considered, 2016 and 2017.

Days after artificial inoculation	Mean *a_w_* level	
	
	2016	2017
14	0.970	0.997
28	0.959	0.986
42	0.947	0.930
56	0.914	0.884


### Aspergillus Section Flavi

The incidence of *AsF* resulted significantly higher in 2017 compared to 2016 (29 vs. 19%) (Table 3). However, no significant differences were noted between the sampling times considered (*P* ≥ 0.05).

**Table 3:** Analysis of variance (ANOVA) of *Aspergillus section Flavi* (*AsF*) and aflatoxin B1 (AFB1) contamination in the 2 years (2016 and 2017), different sampling times (14, 28, 42, and 56 days after inoculation-DAI) and treatments considered in the study (untreated, single and co-inoculum with *Fusarium verticillioides* and *Fusarium graminearum*).

	Incidence of *AsF* (%)			Aflatoxin B_1_ (μg/kg)		
		
Year (A)	^∗∗^		Std Dev	^∗∗^		Std Dev
2016	19.9	B	8.3	150.8	B	13.8
2017	29.4	A	8.9	348.0	A	11.8
**Sampling**	**n.s.**			**^∗∗^**		
**time (B)**						
14 DAI	18.6		8.5	36.5	C	7.9
28 DAI	24.8		9.5	70.4	B	8.0
42 DAI	26.3		9.2	278.8	A	13.3
56 DAI	26.8		8.1	510.2	A	13.9
**Thesis (C)**	**^∗∗^**			**^∗∗^**		
Untreated	6.2	B	1.9	1.5	C	2.3
*A. flavus*	32.9	A	8.4	319.7	B	13.4
*A. flavus + F. verticillioides*	23.1	A	4.5	236.6	B	9.6
*A. flavus + F. graminearum*	34.3	A	11.3	377.5	A	8.5
AxB	^∗^			^∗^		
BxC	n.s.			^∗∗^		
AxC	n.s.			^∗^		
AxBxC	n.s.			^∗^		


*n.s., not significative; ^∗^*P* ≤ 0.05; ^∗∗^*P* ≤ 0.01; Std Dev, standard deviation.*

As expected, significant differences in *AsF* incidence were found between untreated and treated ears (*P* ≤ 0.01), with the former significantly lower, but no significant differences were found between ears artificially inoculated only with *A. flavus* or co-inoculated with *Fusarium* spp (Table 3).

**FIGURE 2:**
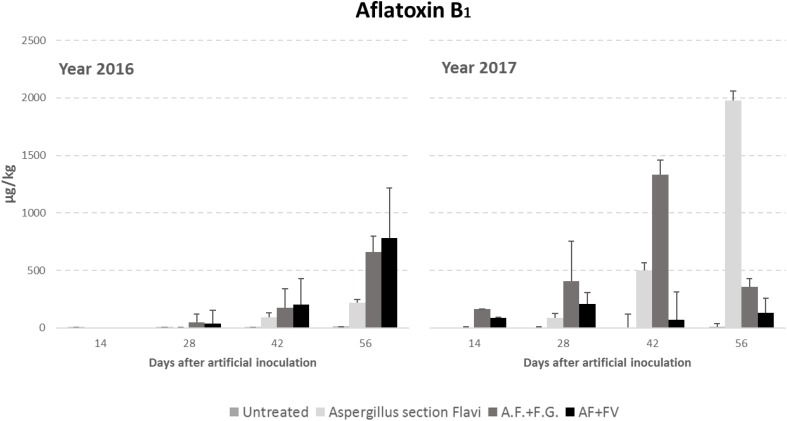
Aflatoxin B1 content in maize ears without artificial fungal inoculation (untreated) and artificially inoculated with *Aspergillus flavus, A. flavus* and *F. verticillioides* (AF+FV) or *A. flavus* and *Fusarium graminearum* (AF+FG) collected at different sampling times during the growing season in years 2016 and 2017.

Regarding AFB1 production, all the factors considered significantly affected kernel contamination (*P* ≤ 0.01) (Table 3). In particular, as well as for fungal incidence, year 2017 resulted as having a contamination by AFB1 double that of 2016 (348 vs. 151 μg/kg); in particular, AFB1 gradually increased during the growing season reaching a maximum after 42 DAI (Table 3). Significant differences were also found between the treatments considered; interestingly, the highest AFB1 production was obtained in the case of co-inoculum with *F. graminearum* (Table 3). Considering the interaction between year, sampling time and inoculation thesis, the impact of co-inoculation with other mycotoxigenic fungi on AFB1 production was the highest in 2016 when, at all sampling times considered, co-inoculated ears resulted having AFB1 content higher than ears inoculated with only *A. flavus* (Figure 2). In year 2017, this trend was obtained only up to 42 days after artificial inoculation; at harvest time, the thesis with only *A. flavus* inoculum resulted the most contaminated by AFB1 (Figure 2).

### *Gibberella fujikuroi* Species Complex

The incidence of *Gf*sc in maize kernels was similar in the 2 years considered (*P* ≥ 0.05) while significant differences were found during the growing season (*P* ≤ 0.01) and in the different treatments (*P* ≤ 0.01) considered in the study (Table 4). In particular, the incidence of *Gf*sc increased gradually during the growing season, significantly higher at 56 DAI (Table 4). Ears with artificial inoculation of only *F. verticillioides* resulted the most contaminated by *Gfsc* (48%) while ears co-inoculated with *F. graminearum* or *A. flavus* resulted not significantly different (around 24–25% incidence) (Table 4).

**Table 4:** Analysis of variance (ANOVA) of *Gibberella fujikuroi* species complex (*Gf*sc) and fumonisins (FBs) contamination in the 2 years (2016 and 2017), different sampling times (14, 28, 42, and 56 days after inoculation-DAI) and treatments considered in the study (untreated, single and co-inoculum with *Fusarium graminearum* and *Aspergillus flavus*).

	Incidence of *Gfsc* (%)			Fumonisins (μg/kg)		
		
Year (A)	n.s.		Std Dev	^∗∗^		Std Dev
2016	27.1		16.2	5469.3	B	45.5
2017	22.6		10.8	7732.1	A	27.0
**Sampling**	**^∗∗^**			**^∗∗^**		
**time (B)**						
14 DAI	13.3	C	5.4	612.7	C	21.9
28 DAI	11.1	C	5.4	1875.6	B	22.4
42 DAI	31.9	B	12.9	11482.0	A	5.7
56 DAI	44.0	A	20.9	10556.0	A	7.6
**Thesis (C)**	**^∗∗^**			**^∗∗^**		
Untreated	2.6	C	1.7	484.1	C	19.7
*F. verticillioides*	48.3	A	14.6	9937.0	A	25.3
*F. verticillioides + F. graminearum*	23.9	B	3.6	9030.7	A	20.9
*F. verticillioides* + *A. flavus*	25.5	B	14.3	6236.3	B	43.4
AxB	^∗∗^			^∗∗^		
BxC	^∗∗^			^∗∗^		
AxC	n.s.			^∗^		
AxBxC	^∗∗^			^∗^		


*n.s., not significative; ^∗^*P* ≤ 0.05; ^∗∗^*P* ≤ 0.01; Std Dev, standard deviation.*

Year resulted to have a significant role in FB production with year 2017 resulting more favorable than year 2016 (Table 4). FBs increased during the growing season following the same trend of *Gf*sc incidence; significant differences were found between sampling times and treatments considered (*P* ≤ 0.01). No significant differences were found between ears inoculated with *F. verticillioides* alone and co-inoculated with *F. graminearum* in FBs contamination, which were significantly more contaminated compared to the others (Table 4).

**FIGURE 3:**
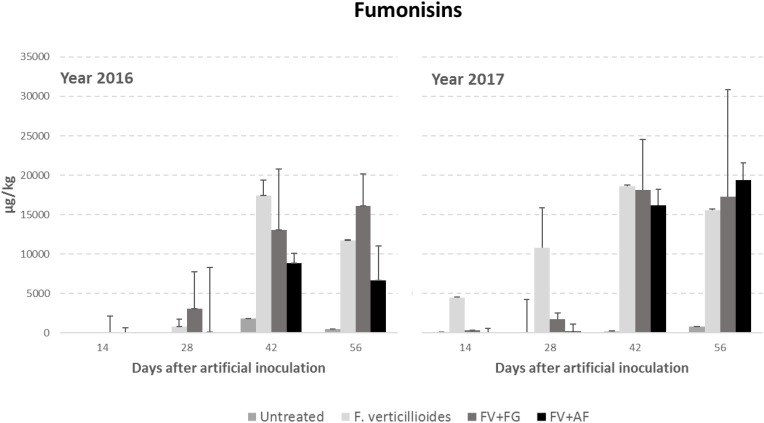
Fumonisin B1+B2 (FBs) content in maize ears without artificial fungal inoculation (untreated) and artificially inoculated with *Fusarium verticillioides* only, *F. verticillioides* and *F. graminearum* (FV+FG) or *F. verticillioides* and *A. flavus* (FV+AF) collected at different sampling times during the growing season in years 2016 and 2017.

The interaction year × sampling time × thesis was significant also for *F. verticillioides*; in particular, the co-inoculum, both with *F. verticillioides* and *A. flavus* resulted in a higher FB content in year 2017 than in the previous year from 42 days after infection (Figure 3).

### *Fusarium graminearum* Species Complex

The incidence of *Fgsc* was not affected by the year (*P* ≥ 0.05) resulting similar and low in both the years considered, without significant differences between sampling times (Table 5). Significant and interesting differences were found between treatments (*P* ≤ 0.01); in particular, ears inoculated with only *F. graminearum* resulted having a lower *Fg*sc incidence compared to those co-inoculated with *F. verticillioides* (Table 5). Contrarily, in the case of co-inoculation with *A. flavus*, the incidence of *Fg*sc resulted very low, not different from untreated ears (Table 5).

**Table 5:** Analysis of variance (ANOVA) of *Fusarium graminearum* species complex (*Fgsc*) and deoxynivalenol (DON) contamination in the 2 years (2016 and 2017), different sampling times (14, 28, 42, and 56 days after inoculation-DAI) and treatments considered in the study (untreated, single and co-inoculum with *Fusarium verticillioides* and *Aspergillus flavus*).

	Incidence of *Fgsc* (%)			Deoxynivalenol (μg/kg)		
		
Year (A)	n.s.		Std Dev	^∗∗^		Std Dev
2016	12.1		8.4	52.2	A	4.8
2017	7.3		4.2	0.0	B	1
**Sampling**	**n.s.**			**^∗∗^**		
time (B)						
14 DAI	8.9		6.7	0.0	B	1
28 DAI	7.6		6.6	0.0	B	1
42 DAI	10.7		6.5	1.7	B	2.5
56 DAI	12.5		6.6	105.2	A	7.5
**Thesis (C)**	**^∗∗^**			**^∗∗^**		
Untreated	0.7	C	0.6	0.0	B	1
*F. graminearum*	11.6	B	6.1	0.0	B	1


*F. graminearum + F. verticillioides*	22.6	A	5.2	111.4	A	8.1
*F. graminearum*+ *A. flavus*	4.8	C	4.3	1.2	B	2.2


AxB	^∗^			^∗∗^		
BxC	n.s.			^∗∗^		


AxC	n.s.			^∗∗^		
AxBxC	n.s.			^∗∗^		




*n.s., not significant; ^∗^*P* ≤ 0.05; ^∗∗^*P* ≤ 0.01; Std Dev, standard deviation.*

Deoxynivalenol was detected only in year 2016 while NIV resulted always absent. The year resulted significant for DON production (*P* ≤ 0.01) and the contamination resulted evident only after 42 days after artificial inoculation reaching the maximum at harvest (Table 5). As well as for fungal incidence, the highest content of DON was found in the thesis where *F. graminearum* was co-inoculated with *F. verticillioides* (Table 5). In the case of co-inoculum with *A. flavus*, DON was found only in traces while it resulted absent in all the other treatments.

### Triple Artificial Inoculation

When the three fungal species (*A. flavus, F. verticillioides*, and *F. graminearum*) were inoculated simultaneously on the same maize ears, year resulted significant only for *AsF* incidence (*P* ≤ 0.05), with almost a double incidence in year 2017 compared to 2016 (Table 6). For *Fusaria*, no significant differences were found between years, but their incidence increased during the growing season, significantly higher at 42 and 56 days after infection compared to the previous sampling time (Table 6). *AsF*, instead, did not show significant differences between sampling times (Table 6).

**Table 6:** Analysis of variance (ANOVA) of Aspergillus section Flavi (*AsF*), Gibberella fujikuroi species complex (*Gfsc*) and Fusarium graminearum species complex (Fgsc) incidence artificially co-inoculated on maize ears and aflatoxin B1 (AFB1), fumonisin B1+B2 (FBs), and deoxynivalenol (DON) contamination in the 2 years (2016 and 2017) and different sampling times (14, 28, 42, and 56 days after inoculation-DAI) considered in the study.

	Incidence of *AsF* (%)			Incidence of *Gfsc* (%)			Incidence of *Fgs* (%)			AFB_1_ (μg/kg)			FBs (μg/kg)			DON (μg/kg)		
						
Year (A)	^∗^		Std Dev	n.s.		Std Dev	n.s.		Std Dev	n.s.		Std Dev	n.s.		Std Dev	^∗^		Std Dev
2016	15.0	B	7.1	26.2		14.7	9.1		4.7	195.5		11.4	9345.7		61.1	9.6	A	3.1
2017	27.9	A	8.1	18.3		11.7	7.0		2.0	121.7		10.6	8882.4		46.5	0.0	B	1.0
**Sampling time (B)**		**n.s.**			**^∗∗^**			**^∗∗^**			**^∗^**			**^∗∗^**			**^∗^**	
14 DAI	21.8		7.3	7.3	B	5.8	1.6	B	2.5	41.9	B	5.0	0.0	B	21.5	0.0	B	1.0
28 DAI	25.3		9.0	3.1	B	4.9	3.1	B	5.0	133.1	AB	8.7	5482.6	A	41.0	0.0	B	1.0
42 DAI	16.9		9.4	32.7	A	16.7	13.8	A	3.4	152.2	AB	11.9	16969.6	A	62.4	3.2	AB	2.2
56 DAI	18.9		5.4	47.6	A	21.7	14.2	A	2.8	306.0	A	15.1	12320.6	A	8.9	17.8	A	4.0
**AxB**		**n.s.**			**^∗^**			**^∗^**			**n.s.**			**n.s.**			**^∗^**	


*n.s., not significative; ^∗^*P* ≤ 0.05; ^∗∗^*P* ≤ 0.01; Std Dev, standard deviation.*

Among considered mycotoxins, only DON resulted to be influenced by the year since it resulted completely absent in year 2017, while both FB and AFB1 contamination resulted similar in both years (Table 6).

All mycotoxins resulted as increasing during the growing season, but in different ways; FBs increased rapidly after 14 days from artificial inoculation and then no significant differences were noted till harvest, while both DON and AFB1 increased slower with the significantly highest content at harvest (Table 6).

## Discussion

The occurrence of the three fungi used in our study has been frequently reported on maize (Pearson and Wicklow, 2006; Mukanga et al., 2010; Krnjaja et al., 2019), however, environmental conditions play a key role in determining their development in field. The years considered in this study showed similar temperatures (<0.5°C difference as mean of the 2-week period considered) except at 28 and 56 DAI. In 2017, temperatures were higher compared to 2016, with 23.3 vs. 28.2 at 28 DAI and 24.4 vs. 25.7 at 56 DAI. Rain and RH were also different, with 2017 resulting dryer than 2016. This greatly influenced *AsF* occurrence, since this fungus is well known to prefer hot and dry conditions (Giorni et al., 2016; Obradovic et al., 2018). As a result, both *AsF* incidence and AFB1 production were higher in the drier year (2017); contrarily, significant differences between years were not noticed for Fusaria incidence. This was probably due to differences in rain fallen in the 2 years considered; in fact, Fusarium Head Blight (FHB) is strongly dependent on specific weather condition (Doohan et al., 2003), mainly rainfall and temperature. A previous study underlined that a monthly rainfall sum of 113.9 mm and a monthly average temperature of 15.5°C are the best conditions for FHB occurrence (Wenda-Piesik et al., 2017).

The efficacy of the initial inoculation was confirmed by the incidence of the three fungal species found on maize kernels, even with differences due to weather conditions registered in each year. During the maize growing season, differences in fungal incidence were found only for Gfsc that increased up to harvest time while *AsF* and *Fgsc* had a very similar incidence for the whole season. This was probably due to *F. graminearum* ability to rapidly colonize and infect different parts of the plant, as already demonstrated in wheat (Mudge et al., 2006). Interestingly, DON production followed the same trend of the fungus, increasing during the growing season but, differently from *F. graminearum* incidence, DON was detected only in 2016 demonstrating, once again, how strictly is dependent from weather conditions (Doohan et al., 2003) and in particular to rain occurrence during the final maize ripening period (Blandino et al., 2017). Even FBs and AFB_1_ increased during the growing season, reaching the maximum from 42 days after artificial inoculation.

This different behavior between fungal growth and mycotoxin production has already been found in other studies, in particular no positive correlation was found between *A. flavus* and *F. verticillioides* in maize while significant and positive correlation was found between their relative mycotoxins (AFB1 and FBs, respectively; Obradovic et al., 2018). Comparable fungal incidence can result in significantly different AFB1 and FB contamination in maize during the growing season due to different weather conditions (Obradovic et al., 2018, Krnjaja et al., 2019).

Surely, also the host played a role; in particular, the *aw* of maize kernels was reported to be particularly important. Only when maize *aw* becomes lower than 0.95, the highest increment in AFB1 was noted in both years considered, confirming results obtained in a previous study (Giorni et al., 2016).

Even if differences in fungal incidence were not statistically significant, it is important to note that, in the case of co-inoculum, *A. flavus* resulted as being more affected by *F. verticillioides* than by *F. graminearum*; the incidence of *AsF* showed a 10% decrease when co-inoculated with *F. verticillioides* (23 vs. 33%), while, in the case of co-occurrence with *F. graminearum, AsF* incidence was around 34%, similarly to single *A. flavus* inoculated ears. This behavior is confirmed also for AFB1 production obtaining the lowest content in case of *F. verticillioides* co-occurrence. A previous study (Giorni et al., 2009) underlined the ability of *A. flavus* and *F. verticillioides* to grow at the same time on maize since they usually occupy different niches regarding carbon sources; however, in certain environmental conditions, one fungal species can become dominant on the other. In particular, *F. verticillioides* seems to be dominant, because it is able to use more carbon sources, at the lowest temperatures (15°C) and the highest *aw* levels (>0.95 *aw*) while *A. flavus* becomes dominant, which means more efficient and quicker in using carbon sources, at higher temperatures (>25–30°C) and dry conditions (0.87*aw*) (Marin et al., 1995; Sanchis and Magan, 2004; Giorni et al., 2009). This impacts also on mycotoxin production.

Comparing our results with weather conditions observed during the growing season, it is interesting to note how mean temperatures result always conducive for both AFB1 and FB production, being in both years ≥ 24°C; however, a_w_ measured in maize ears when AFB_1_ and FBs resulted the highest were quite far from optimal levels reported for mycotoxin production (a_w_ ≤ 0.93 vs. a_w_ ≥ 0.98). This seems to confirm the discrepancy in fungal behavior found between *in vitro* and *in field* studies, as previously underlined (Payne et al., 1988; Battilani et al., 2008). In particular, a_w_ ≤ 0.95, occurring in field during maize ripening, was shown as the most suitable condition for a rapid aflatoxin accumulation *in field*, switching the fungal metabolism in favor of high AF production, the opposite observed in *in vitro* conditions (Giorni et al., 2016). It is interesting to note that AFB1 production was the highest in the case of co-occurrence with *F. graminearum*. This probably means that *F. graminearum* can interfere with *A. flavus* development, increasing its stress with competing significantly; consequently, AFB1 production resulted significantly higher compared to the other studies’ conditions. No previous reports were found regarding specific interaction between these two fungi either in field nor *in vitro*. It is therefore a topic to be studied more deeply.

Regarding *Gfsc*, maximum incidence was found only in the case of single inoculation; when co-inoculated with either *F. graminearum* or *A. flavus, Gfsc* showed an incidence reduction of almost 50%. The same did not happen with FB production which resulted reduced only in the case of co-inoculation with *A. flavus*. In a previous study, using data from *in vitro* trials, it has been demonstrated a good correlation among temperature, a_w_ and FBs production by *F. verticillioides* (Marín et al., 1999), however data showed a good fit only in a limited range of temperatures (15–30°C) and only at 0.97 a_w_, suggesting that FBs production could be possible only in a limited environmental condition range. In field, fungal metabolism may change, as suggested for *A. flavus* (Giorni et al., 2016), in order to have adaptation to unfavorable environmental condition or limited nutritional availability, like those occurring with fungal co-existence.

Interestingly, *Fgsc* showed a different behavior having its highest incidence in the case of co-inoculum with *F. verticillioides*, even higher than single inoculum (23 vs. 12%) while, in the case of co-inoculum with *A. flavus* its occurrence decreased enormously, with less than 5% incidence. This means that *F. graminearum* was enhanced by the co-occurrence with *F. verticillioides* and it was very able to compete with this fungus for space and nutrients as previously found (Velluti et al., 2001). High competitivity between these two fungi was observed also in the case of mycotoxin production; in fact, the highest production of both FBs and DON was achieved in the case of their co-inoculum, while in the case of co-inoculum with *A. flavus* mycotoxin production decreased for both fungi. This confirms the findings of previous studies where the co-occurrence of *F. graminearum* with *F. verticillioides* was able to increase FB1 production in the case of specific environmental and substrate conditions (25°C and 0.98 *aw*) and the growth of *F. graminearum* resulted greatly stimulated in the case of co-occurrence with *F. verticillioides* (Velluti et al., 2001). Moreover, it seems that mycotoxin production can play a role in fungal interactions; in particular, in a previous study, low DON concentrations resulted as acting as a signal for competing species inducing a higher FB1 production able to significantly reduce *F. graminearum* growth (Dawidziuk et al., 2016).

When the two fungal species were inoculated at the same time on maize ears it was interesting to note a similar behavior as in the case of single fungal presence or the co-occurrence of two species. In particular, *AsF* maintained constant its incidence during the growing season while Fusaria species reached the maximum only at 42 DAI. Regarding mycotoxins, AFB1, FBs, and DON increased during the growing season as previously reported even if DON production resulted limited and slower.

This is one of the first studies conducted in field on the dynamic of multi-mycotoxins and the co-occurrence of fungi in artificially inoculated maize. Several studies took into consideration the occurrence of different mycotoxins only at harvest time, without considering the fungal dynamics due to their interaction on maize plants during the growing season (DongHo et al., 2017; Adekova et al., 2018). To understand these fungi interactions is instead fundamental in order to predict the effect on mycotoxin production and, as a result, have a correct risk assessment, especially in the challenging scenario of climate change.

Data obtained in this study can be considered a starting point to define the role of environmental factors and competition between fungal species in maize in field. They contribute to understand fungal dynamics in case of fungi co-occurrence and their impact on mycotoxins, but this is a multifaced answer that needs to be studied deeper and further confirmed.

## Contribution to the Field Statement

Mycotoxin co-occurrence is increasingly stressed due to the relevant impact of climate change on fungi interaction. Extreme events occur during the growing season of different crops, maize included, and they alternatively favor fungi with different ecological needs. Consequently, diverse mycotoxins can be detected in agricultural products coming from the same field. The dominance of one fungus over the others is difficult to predict, especially if the whole season is considered. Therefore, this paper contributes to add knowledge on this topic.

In particular, several studies were previous conducted, both *in vitro* and *in field*, with the inoculum of single mycotoxigenic species trying to understand the ecological conditions necessary for their development and for mycotoxin production. However, these conditions can become different in the case of more fungal species present in the same matrix because of strong competition influencing also their mycotoxin production.

This is one of the first studies on artificial inoculation in maize fields with more than one mycotoxigenic fungal species. The results obtained contribute to the understanding of fungal dynamics in the case of co-occurrence and its impact on mycotoxin co-occurrence.

## Data Availability

All datasets generated for this study are included in the manuscript and/or the supplementary files.

## Author Contributions

PB designed and supervised the research, and assisted in manuscript writing and revision. PG performed the trial, analyzed the data, and wrote the manuscript. TB analyzed the samples and assisted in manuscript development. All authors wrote the manuscript and approved the final version of the manuscript.

## Conflict of Interest Statement

The authors declare that the research was conducted in the absence of any commercial or financial relationships that could be construed as a potential conflict of interest.
